# The microbiological characteristics of *Staphylococcus aureus* isolated from patients with native valve infective endocarditis

**DOI:** 10.1080/21505594.2019.1685631

**Published:** 2019-11-13

**Authors:** Chung-Jong Kim, Kyoung-Ho Song, Pyoeng Gyun Choe, Wan Beom Park, Eu Suk Kim, Kyoung Un Park, Nam Joong Kim, Kyung-Hwa Park, Yee Gyung Kwak, Shinhye Cheon, Hee-Chang Jang, Young Keun Kim, Sun Hee Lee, Sung-Min Kiem, Shinwon Lee, Hong Bin Kim, Myoung-don Oh

**Affiliations:** a Department of Internal Medicine, Ewha Womans University Seoul Hospital, Seoul, Republic of Korea; b Department of Internal Medicine, Seoul National University Bundang Hospital, Seongnam, Republic of Korea; c Department of Internal Medicine, Seoul National University College of Medicine, Seoul, Republic of Korea; d Department of Internal Medicine, Seoul National University Hospital, Seoul, Republic of Korea; e Department of Laboratory Medicine, Seoul National University Bundang Hospital, Seongnam, Republic of Korea; f Department of Infectious Diseases, Chonnam National University Hospital, Gwangju, Republic of Korea; g Department of Internal Medicine, Inje University Ilsan Paik Hospital, Goyang, Republic of Korea; h Division of Infectious Diseases, Department of Internal Medicine, Chungnam National University School of Medicine, Daejon, Republic of Korea; i Department of Internal Medicine, Chonnam National University Hwasun Hospital, Hwasun, Republic of Korea; j Department of Internal Medicine, Yonsei University Wonju College of Medicine, Wonju, Republic of Korea; k Department of Internal Medicine, Pusan National University School of Medicine and Medical Research Institute, Pusan National University Hospital, Busan, Republic of Korea; l Department of Internal Medicine, Inje University Haeundae Paik Hospital, Busan, South Korea; m Department of Internal Medicine, Daegu Fatima Hospital, Daegu, South Korea

**Keywords:** *Staphylococcus aureus*, bacteremia, infective endocarditis, fibronectin, single-nucleotide polymorphism, virulence

## Abstract

The microbiological characteristics of *Staphylococcus aureus* causing infective endocarditis (IE) have not been investigated thoroughly. We compared the characteristics of *S. aureus* isolates from patients with and without IE. Cases of *S. aureus* bacteremia (SAB) were collected from 10 hospitals over 7 years. Cases of native valve IE were matched with non-IE controls according to the following criteria: central-line-associated infection, community-acquired infection, methicillin susceptibility, and if possible, the primary site of infection. Genes coding virulence factors were analyzed using multiplex polymerase chain reactions. Fibrinogen and fibronectin-binding properties were assessed using *in vitro* binding assays. The fibronectin-binding protein A gene (*fnbpA*) was sequenced. Of 2,365 cases of SAB, 92 had IE. After matching, 37 pairs of *S. aureus* isolates from the IE cases and non-IE controls were compared; *fnbpA* was detected in 91.9% of the IE isolates and 100% of the non-IE isolates (*p* = 0.24). While the fibrinogen binding ratio was similar (1.07 ± 0.33 *vs*. 1.08 ± 0.26, *p* = 0.89), the fibronectin-binding ratio was significantly higher in the IE-group (1.31 ± 0.42 *vs*. 1.06 ± 0.31, *p* = 0.01). The proportions of major single-nucleotide polymorphisms in *fnbpA* were as follows: E652D (2.9% *vs*. 2.7%), H782Q (65.6% *vs*. 60.6%), and K786N (65.6% *vs*. 72.7%). The fibronectin-binding ratio was positively correlated with the number of SNPs present in IE cases (*p* < 0.001) but not in the non-IE controls (*p* = 0.124). Fibronectin-binding might play a key role in SAB IE. However, the degree of binding may be mediated by genetic variability between isolates.

## Introduction

*Staphylococcus aureus* causes a variety of infections ranging from soft tissue infections to more serious conditions, like infective endocarditis (IE) [1,2]. IE is a very serious infection because of potential damage to cardiac structures, along with embolic events in major organs such as the central nervous system, resulting in high mortality and poor clinical outcomes [1–4]. Known clinical risk factors for IE include the presence of intracardiac devices, infections not associated with a central catheter, presence of infectious spondylitis, and prolonged bacteremia [2,5].

Although the precise pathogenesis of IE is unknown, studies suggest that adhesion of bacteria to the endothelial layers is an important step in the pathogenesis of IE [2,6]. Until recently, endothelial damage was considered an integral step in the pathogenesis of IE; however, it has been shown that some bacteria, especially *S. aureus*, can cause IE, even in undamaged endothelial tissues [7–9]. This suggests that the ability of *S. aureus* to attach to undamaged endothelial cells could be the key mechanism in *S. aureus* IE. This observation has led many to speculate that pathogen-associated factors may play more a central role in the development of IE than previously thought [10,11].

*Staphylococcus aureus* expresses a wide range of virulence factors, including microsomal surface component recognizing adhesive matrix molecule family (MSCRAMM) and numerous secretory toxins [12]. MSCRAMM mediates the adhesion between *S. aureus* and matrix materials such as fibrinogens, fibronectins, and collagens [13,14]. Among these various materials, fibrinogen and fibronectin are important components of the extracellular matrix of endothelial cells. Fibronectin is a direct target of *S. aureus*, enabling attachment and internalization of bacteria into endothelial cells [15,16]. Because binding of fibronectin by *S. aureus* is solely mediated by fibronectin-binding protein (fnbp) A or B, the capacity of fnbp to bind fibronectin may play an important role in the development of IE. However, there have been few comprehensive evaluations of the microbiological factors affecting the development of *S. aureus* IE, especially using clinical isolates of endocarditis.

In this study, we compared the characteristics of *S. aureus* isolated from patients with IE and clinically matched non-IE to identify microbiological factors affecting the development of IE. Furthermore, fnbp and its relationship with IE was also evaluated.

## Material and methods

### Study population

Medical records and bacterial isolates from *S. aureus* bacteremia (SAB) cases were collected from 10 Korean hospitals between 2009 and 2015. All cases were registered prospectively at each study hospital, with bacterial isolates archived for further analysis. Patient age (≤15 years old), polymicrobial bloodstream infection, and cases of obvious contamination were excluded from the SAB case records. Patients who had intracardiac devices, such as prosthetic valves or cardiac pacing devices, were excluded from the analyses.

### Clinical data collection

Clinical data of enrolled cases were recorded using an electronic case record form operated by a central institution. Collected variables included demographic data, presence of an intracardiac device, date, and location of onset, portal of entry of bacteria, site of infection, use of a central venous catheter, and presence of intracardiac infections such as paravalvular abscess or vegetation.

### Definitions

Definitions used in this study are similar to those in previously [17]. Hospital-onset infection was defined as bacteremia that occurred 48 h after admission. Community-onset infection was defined as bacteremia that occurred within 48 h of admission. Community-acquired infection was defined as cases who had no healthcare-associated risk factors at the bacteremia onset among cases of community-onset infection. Healthcare-associated risk factors included 2 or more days of hospital admission in the year prior to the current admission, living in a health-care facility, intravenous drug administration in the 30 days prior to the current admission, and renal replacement treatment in the 30 days prior to the current admission. Central line-associated bloodstream infection (CLABSI) was defined as bacteremia in cases where a central venous catheter was either used or removed within 48 h of bacteremia onset, without any other obvious focus of infection. Prolonged bacteremia was defined as persistent blood culture positivity despite ≥7 days of appropriate antibiotic treatment. The presence of IE was defined according to the modified Duke criteria.

### Classification of IE and non-IE groups

Cases of SAB were categorized into the IE, non-IE, and unclassified groups as follows. Cases were categorized in the IE group if transthoracic or transesophageal echocardiography revealed an intracardiac infection, such as valvular vegetation or paravalvular abscess. If both transthoracic and transesophageal echocardiography revealed no evidence of an intracardiac infection, then the cases were categorized in the non-IE group. If only transthoracic echocardiography was performed to exclude intracardiac infection, then the cases were place in the unclassified group, and were excluded from further analysis. For case–control matching, the non-IE group was selected based on a 1:1 match with the IE group according to the following criteria: i) CLABSI, ii) community-acquired infection, and iii) methicillin susceptibility. When there were multiple possible control patients, then primary site of infection was also considered. Non-matched IE cases were not included in further analyses.

### Preparation of S. aureus isolates

*S. aureus* isolates were collected at each of the participating hospitals and stored at −70°C using Microbank (Pro-Lab Diagnostics, Austin, TX, USA). Samples were then transferred to a central laboratory facility. Frozen bacterial isolates were thawed and resuspended in Trypticase soy broth (TSB), and grown overnight. After overnight growth, 1 mL of bacterial suspension was added to 9 mL of fresh TSB and grown to mid-log phase. Bacteria were then collected by centrifugation, and resuspended in fresh TSB medium in each experiment.

### Preparation of endothelial cells

Human umbilical vein endothelial cells (HUVECs) (Cambrex, MD) were used for cell culture experiments. Cells were grown to confluence in 12-well plates at 37°C in antibiotic-free EGM-2 media containing 10% fetal bovine serum and other growth factors. All experiments were performed with cells that had been passaged between four and eight times.

### Analyses of virulence factors of S. aureus isolates

Virulence factors of *S. aureus* were assessed by multiplex polymerase chain reaction (PCR), as described previously [18]. We assessed the virulence factors associated with cell adhesion, such as *clfA, clfB, fnbpA, fnbpB, sdrC, sdrD, sdrE, bbp, cna, ebpS*, and *map*, and excretory toxins such as *sea, seb, sec, sed, see, seg, seh, sei, sej, tst, eta*, and *etb*. Genomic DNA was extracted from *S. aureus* using a QIAGEN DNA extraction kit (QIAGEN, Hilden, Germany).

### Bacterial adhesion to purified fibronectin and fibrinogen

Bacterial adhesion to extracellular matrix (ECM) materials was assayed using purified fibronectin and fibrinogen. A total of 30 µL of ECM material (human fibronectin, 30 µg/mL in TBS or fibrinogen 50 µg/mL in PBS; both Sigma Aldrich) was loaded onto a 96-well plate and incubated overnight at 4°C. The same amount of PBS was used as a negative control. *S. aureus* laboratory strains 8325–4 (FnBP-A + B+) and DU 5883 (FnBP-A − B−) were used as standard controls. After overnight incubation, each well was flooded with 1% bovine serum albumin (BSA) and incubated at 37°C for 20 min. After incubation, 200 µL of bacterial suspension (5 × 10^7^ CFU/mL) was added to each well and incubated for 60 min at 37°C. Wells were then washed with PBS and attached bacteria were adhered using glutaraldehyde for 2 h at 4°C. Attached bacteria were stained with crystal violet and assayed at an optical density (OD) of 570 nm. All tests were performed in triplicate.

The degree of attachment is presented as the ratio of the OD of experimental isolates relative to a standard control (strain 8325–4). To avoid bias due to inter-isolate variability, we also assessed the degree of attachment using the OD of experimental materials relative to PBS.

### HUVEC internalization

Invasion of endothelial cells was assessed by the internalization of isolates into HUVECs, as described previously [6,19]. Briefly, HUVECs were grown in 12-well plates in antibiotic-free EGM-2 media. A bacterial suspension of 2 mL (1 × 10^6^ CFU/mL) was added to each well and incubated for 1 h at 37°C. After washing with PBS, cells were detached by trypsin and disrupted in distilled water. The number of internalized bacteria was assayed by serial dilution after overnight incubation of the distilled water. All tests were performed in triplicate.

### *Sequencing of* fnbpA

Amino acid polymorphisms in FnbpA were assessed by the sequencing of the variable regions in *fnbpA*. The variable region of FnbpA was amplified using primers fnbpA-F and fnbpA-R. Among the 11 FnBRs, FnBR1 through FnBR4 were sequenced and compared to the standard sequence.

### Statistical analysis and ethics review

The Mann–Whitney *U*-test was used to compare continuous variables, and the chi-square test was used to compare categorical variables. If the expected number of instances of a given outcome was <5, Fisher’s exact test was used. This study was approved by the institutional review boards of Seoul National University Hospital (H-1608-142-787) and Seoul National University Bundang Hospital (B-1905-540-403).

## Results

### Baseline characteristics

**Figure 1.**
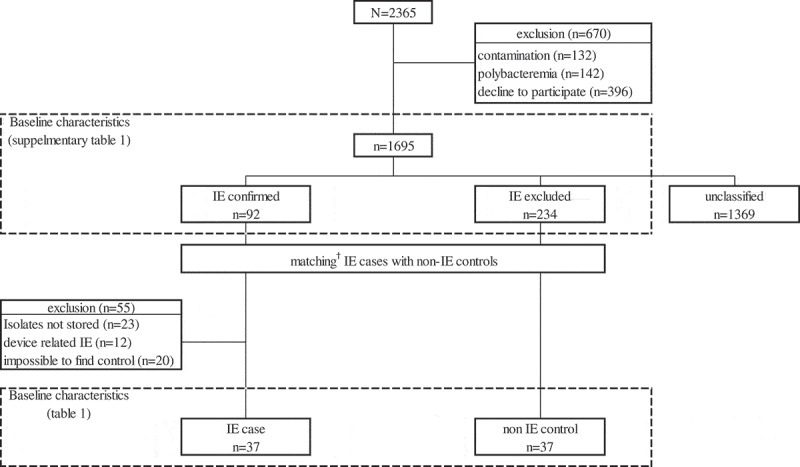
Flow of case selection. † Matching criteria were i) central-line-associated bloodstream infection, ii) community-onset infection, iii) methicillin susceptibility, and iv) primary site of infection. Note. IE, infective endocarditis.

Among the 2,365 SAB cases who were identified during the study period, 1,695 were screened to find the IE and non-IE groups after excluding the patients with possible contamination (n = 132), polymicrobial bacteremia (n = 142), or who declined to participate in the study (n = 396). Of these eligible cases, IE was identified in 92 cases (IE group, 5.4% of the 1,695 cases), and SAB without IE was identified in 234 cases (non-IE group, 13.8%), among the 326 cases in whom both the transthoracic and transesophageal echocardiography was performed. Supplementary Table 1 shows the baseline characteristics of the IE and non-IE groups. After matching IE cases with non-IE controls at a 1:1 ratio, 37 pairs of IE and non-IE cases were selected for the final analysis (Figure 1).

Fifty-five cases of SAB IE were excluded from the microbiological analysis either because the bacteria were not stored (n = 23), the endocarditis was device related (n = 12), or no non-IE controls could be identified (n = 20). Table 1 shows the baseline characteristics of the selected pairs.

**Table 1.** Baseline characteristics and demographics of matched* cases with *Staphylococcus aureus* bacteremia.

Variables	Native valve Endocarditis group (*n* = 37)	Non-endocarditis group (*n* = 37)
**Methicillin-resistant *S. aureus***	17 (45.9%)	17 (45.9%)
**Community-acquired infection**	9 (24.3%)	9 (24.3%)
**Cardiac prosthesis**	0	0
**SOFA score** ≥ **6**	18 (48.6%)	10 (27.0%)
**Prolonged bacteremia**	13 (35.1%)	13 (35.1%)
**Primary site of infection**		
Primary bacteremia	0	11 (29.7%)
Central line-associated infection	5 (13.5%)	5 (13.5%)
Endovascular other than endocarditis	6 (16.2%)	0
Infective endocarditis	12 (32.4%)	0
Vertebral osteomyelitis	2 (5.4%)	6 (16.2%)
Bone or joint infection other than vertebral osteomyelitis	6 (16.2%)	4 (10.8%)
Skin and soft tissue infection	3 (8.1%)	7 (18.9%)
Lung	0	1 (2.7%)
Others	3 (8.1%)	3 (8.1%)

SOFA, sequential organ failure assessment

* matching criteria: i) central line associated infection, ii) community-acquired infection, iii) methicillin susceptibility, and iv) if possible, primary site of infection.

### Analysis of virulence factors

*Staphylococcus aureus* genes encoding various virulence factors were analyzed by multiplex PCR (Table 2). The proportion of isolates harboring the virulence genes *fnbpA*, and *fnbpB* were similar between the two groups, with all isolates encoding *clfA* and *clfB*. The proportion of isolates harboring virulence genes related to MSCRAMMs was also similar between the two groups. The proportion of isolates harboring *sea* was significantly higher in the non-IE than IE group (5.4% vs. 21.6%, *p* = 0.041). Other genes encoding exotoxins were similarly distributed between the two groups.

**Table 2.** Distribution of virulence factor genes in *Staphylococcus aureus* isolates from patients with and without endocarditis.

Virulence factor gene	*S. aureus* from Endocarditis group (*n* = 37)	*S. aureus* from Non endocarditis group (*n* = 37)	P-value
*clfA*	37 (100%)	37 (100%)	N/A
*clfB*	37 (100%)	37 (100%)	N/A
*fnbpA*	34 (91.9%)	37 (100%)	0.240*
*fnbpB*	4 (10.8%)	5 (13.5%)	1.000*
*sdrC*	34 (91.9%)	33 (89.2%)	1.000*
*sdrD*	26 (70.3%)	26 (70.3%)	1.000
*sdrE*	31 (83.8%)	32 (86.5%)	0.844
*bbp*	36 (97.3%)	37 (100%)	1.000*
*cna*	10 (27.0%)	15 (40.5%)	0.219
*ebpS*	28 (75.7%)	27 (73.0%)	0.790
*map*	2 (5.4%)	2 (5.4%)	1.000*
*sea*	2 (5.4%)	8 (21.6%)	0.041
*seb*	1 (2.7%)	1 (2.7%)	1.000*
*sec*	9 (24.3%)	7 (18.9%)	0.572
*sed*	0	1 (2.7%)	1.000*
*see*	0	0	N/A
*seg*	26 (70.7%)	21 (56.8%)	0.227
*seh*	1 (2.7%)	3 (8.1%)	0.615*
*sei*	26 (70.3%)	21 (56.8%)	0.227
*sej*	0	1 (2.7%)	1.000*
*tst*	11 (29.7%)	10 (27.0%)	0.797
*eta*	1 (2.7%)	0	1.000*
*etb*	0	0	N/A
*sarA*	37 (100%)	37 (100%)	N/A

N/A, Not applicable.

* Statistical analyses were performed with Fisher’s exact test.

### Extracellular matrix binding and cell invasion

The fibrinogen and fibronectin-binding properties are shown in Table 3. While fibrinogen binding was similar between IE and non-IE groups (1.07 *vs*. 1.08, *p* = 0.885), fibronectin-binding was significantly higher in the IE group (1.31 *vs*. 1.06, *p* = 0.007). Statistical differences were also maintained if the ratio was calculated based on PBS binding rather than binding to reference standard isolates (Supplementary Table 2). HUVEC internalization of bacteria was consistent across the IE and non-IE groups (7.0 × 10^6^ CFU *vs*. 6.8 × 10^6^ CFU, *p* = 0.665).

**Table 3.** Physiological properties of *Staphylococcus aureus* isolated from patients with or without infective endocarditis.

Physiological property	Endocarditis group (*n* = 37)	Non endocarditis group (*n* = 37)	P-value
Fibrinogen binding ratio	1.07 (± 0.33)	1.08 (± 0.26)	0.885
Fibronectin binding ratio	1.31 (± 0.42)	1.06 (± 0.31)	0.007
Internalization (×10^6^ CFU/mL)	7.0 (± 9.4)	6.8 (± 7.7)	0.665

CFU, colony forming unit.

### Single-nucleotide polymorphisms of fnbpA

**Figure 2.**
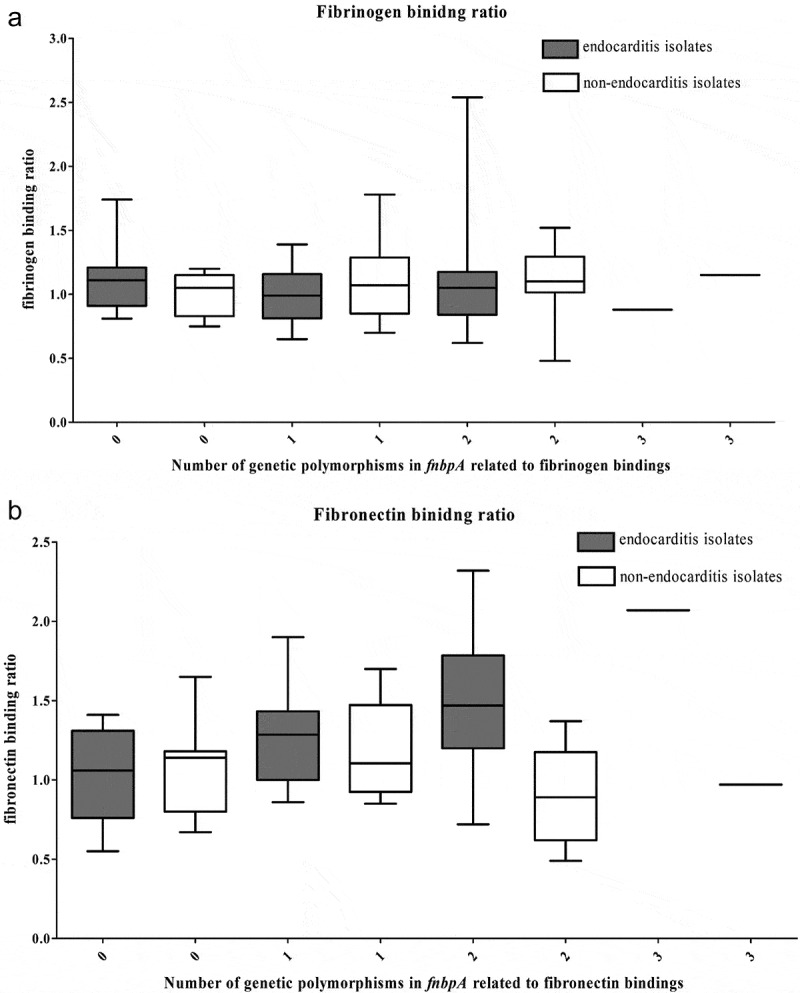
Correlations of the (a) number of genetic polymorphisms in the *fnbpA* gene and physical properties and (b) fibronectin-binding ratio of 37 *Staphylococcus aureus* isolates from native valve infective endocarditis cases and 37 *S. aureus* isolates from matched non-infective endocarditis bacteremia controls.

Next, we analyzed single-nucleotide polymorphisms in *fnbpA*. Table 4 shows the frequently found polymorphisms. The distributions of E652D, H782Q, and K786N were similar between the two groups. No other polymorphisms were identified that were more frequently observed in the IE group. While individual SNPs did not vary, the fibronectin-binding ratio did increase in IE cases in relation to the number of SNPs present, a phenomenon not seen in the non-IE group (Figure 2).

**Table 4.** Distribution of genetic polymorphisms *in fnbpA* gene of *Staphylococcus aureus* isolated from patients with or without endocarditis.

Amino acid change in FnbpA	*S. aureus* isolated from Endocarditis patients (*n* = 37)	*S. aureus* isolated from Non endocarditis patients (*n* = 37)	P-value
**Single amino acid variation**			
E652D ^a^	1 (2.9%)	1 (2.7%)	1.000
H782Q ^b^	21 (65.6%)	20 (60.6%)	0.675
K786N/I ^b^	21 (65.6%)	24 (72.7%)	0.535
H818Q ^b^	15 (46.9%)	14 (42.4%)	0.718
T826N ^b^	16 (50.0%)	14 (42.4%)	0.540
S839N ^b^	25 (78.1%)	28 (84.8%)	0.485
V668I ^a^	5 (14.3%)	4 (10.8%)	0.723
S678I ^a^	10 (28.6%)	9 (24.3%)	0.683
V698I ^a^	33 (94.3%)	33 (89.2%)	0.434
N750D ^b^	4 (12.5%)	3 (9.1%)	0.708
P779T ^b^	0	1 (4.2%)	0.462
N788D ^b^	24 (75.0%)	27 (81.8%)	0.504
K797E ^b^	2 (6.2%)	1 (3.0%)	0.613
H804Q ^b^	29 (90.6%)	29 (87.9%)	1.000
N846S ^b^	18 (56.2%)	14 (42.4%)	0.265
A5941 ^a^	10 (28.6%)	9 (24.3%)	0.683
V647L ^a^	9 (25.7%)	5 (13.5%)	0.191
G656D ^a^	10 (28.6%)	9 (24.3%)	0.683
V662I ^a^	5 (14.3%)	4 (10.8%)	0.732
H706Q ^a^	5 (14.3%)	4 (10.8%)	0.732
I808L ^b^	5 (16.1%)	4 (12.1%)	0.729
**Combinations of single amino acid variations**			
E652D + H782Q + K786N	1 (3.0%)	1 (3.0%)	1.000
H782Q + K786N	16 (48.5%)	14 (42.4%)	0.621
K786N + N788D	17 (51.5%)	19 (57.6%)	0.621
H782Q + N788D	20 (60.6%)	18 (54.5%)	0.619
H782Q + K786N + N788D	14 (42.4%)	12 (36.4%)	0.614
H782Q + K786N + N788D + H818Q	5 (15.2%)	4 (12.1%)	1.000
H782Q + K786N + H818Q	7 (21.2%)	5 (15.2%)	0.751

^a^ Number of isolates: 35 in the endocarditis group and 37 in the without endocarditis group.

^b^ Number of isolates: 33 in the endocarditis group and 33 in the without endocarditis group.

## Discussion

This study evaluated the microbiological characteristics of *S. aureus* isolates from patients with IE compared with isolates from matched non-IE patients. We observed that fibronectin-binding was significantly higher in the *S. aureus* isolated from IE patients, while binding to fibrinogen and internalization into endothelial cells were similar between the two groups. As fibronectin is known to play a role in the attachment of microorganisms to vascular endothelial cells [15,16], the relative affinity of *S. aureus* isolates to fibronectin may represent an important risk factor for IE. However, the proportion of isolates harboring genes encoding fibronectin-binding proteins did not differ between the groups, suggesting that the presence of a virulence gene itself does not affect the ability to initiate IE. Rather, we found a strong correlation between the total number of SNPs encoding fibronectin-binding proteins and the ability to bind fibronectin among IE isolates.

The association between fibronectin and IE has been described previously [20,21]. Fibronectin is known to play a role in both the attachment of bacteria to endothelial cells and the internalization of bacteria after the initial binding. Kerdudou *et al*. reported that deletion of the *fnbp* gene reduced the attachment of bacterial isolates to endothelial cells [22], while deletion of both *fnbpA* and *fnbpB* further reduced the ability of bacteria to attach to endothelial cells [23]. Despite these observations, the presence of the *fnbp* gene itself may not be sufficient to cause IE. An analysis of 12 IE and 10 non-IE SAB isolates revealed no differences in *fnbpA* and *fnbpB* expression between groups [24]. These results were further supported by a larger study assessing 72 IE and 54 non-IE isolates, revealing no differences in protein expression between groups [10]. In these studies, over 90% of isolates harbored the *fnbp* genes. In our study, we also found that the distribution of genes related to fibronectin-binding did not differ between IE and non-IE isolates.

Many studies have suggested that fibronectin and fnbp are related to the development of IE. Our results showed that, while the presence of the *fnbp* gene alone was not sufficient for the pathogenesis of IE, the function of this protein may be central to the development of IE. Among the potential mechanisms underlying this phenomenon, there are two basic possibilities in terms of the functionality of fnbp expression. The first possibility is the presence of genes regulating the expression of *fnbp*. Previous studies have shown that the absence of *sarA*, a gene known to regulate fnbp expression, reduced fibronectin adhesion *in vitro* [25], as well as decreasing the incidence of IE in animal models [26]. In our study, all isolates tested positive for *sarA*, regardless of group; however, the expression of this gene may be different for each strain. Additional studies will be necessary to determine the extent to which gene expression differences play a role in disease pathogenesis. The second possibility is that the binding affinity of fibronectin is altered by genetic polymorphisms in *fnbp*. Many of these polymorphisms may alter the resulting amino acid sequence, which could affect the binding forces between bacteria and endothelial components, altering the virulence.

*Staphylococcus aureus* binds to fibronectin through interactions mediated by the B, C, and D regions of fnbp [27]. fnbp is composed of 11 fibronectin-binding repeats (FnBRs), of which differences in the amino acid sequence of regions have been shown to affect binding strength [28]. Analysis of *S. aureus* isolated from patients with intra-cardiac devices revealed changes in *fnbp* residues 652, 782, and 766, which were strongly predictive of intracardiac device-related infection [28]. While similar observations have been reported in other studies [29], a recent study of prosthetic joint infection with *S. aureus* found no polymorphic or amino acid sequence differences in the *fnbp* gene [11]. Here, we found that SNPs in previously described loci including E652D, H782Q, K786N, H818Q, T826N, and S839N were not significantly different between the IE and non-IE groups. However, while these SNPs were not significant on an individual basis, isolates possessing a combination of the E652D, H782Q, and K786N substitutions did exhibit increased fibronectin binding, particularly in those with a large number of genetic variations. These results are consistent with those of Lower *et al*., who showed a strong correlation between E652D, H782Q, and K786N substitutions and fibronectin-binding [28]. However, there are probably other factors influencing these changes in binding affinity, as this phenomenon was only observed in IE isolates. Changes in the amino acid sequence have been shown to increase the incidence of IE as a result of increased binding between fnbp and fibronectin. It is assumed that structural changes arising from these amino acid substitutions are mediated by charges in branches, along with alterations in the polarity of individual residues. While individual substitutions may not be sufficient to alter virulence, an accumulation of amino acid changes was associated with greater virulence in this study, with multi-substitution isolates significantly more common within the IE group.

Most previous studies comparing bacteremia between IE cases and non-IE cases did not consider the primary site of the infection [10,24]. However, the origins of infections and community-onset or hospital-onset infection are important clinical factors in the development of IE, and they may act as confounders if not controlled for in comparisons of the two groups. In this study, we tried to compensate for clinically important confounding factors by matching select patients with endocarditis and corresponding control patients based on a relatively large number of *S. aureus* bacteremia cases. Although we had to exclude some cases from the analyses because no matched control was available, we believe that our data more clearly reveal the nature of the *S. aureus* strains that cause IE.

There are some limitations to this research. First, we were unable to assess many of the relevant processes associated with virulence *in vivo*. Many virulence factors such as immune evasion, super-antigen production, and toxin secretion are also important factors for developing IE. In this study, we determined whether these genetic factors were present in the microbes, but did not perform functional evaluations. Second, while we did confirm the presence of virulence factors within the *S. aureus* genome, the expression of these genes was not evaluated.

In conclusion, our study suggests that fibronectin-binding plays a key role in *S. aureus* IE. However, the degree of binding does not appear to be mediated by the presence of the *fnbpA* gene itself but may be mediated by genetic variability among isolates.

## Supplementary Material

Supplemental Material
