# Supplementary material for: The microbiological characteristics of Staphylococcus aureus isolated from patients with native valve infective endocarditis
Source: Virulence. 2019 Nov 13;10(1):948–56. doi: 10.1080/21505594.2019.1685631 (PMC8647854; doi:10.1080/21505594.2019.1685631)
Supplement: Supplemental Material [file KVIR_A_1685631_SM2020.docx]

**Supplementary Table**

**S-table 1. Baseline characteristics and demographics of cases with** ***Staphylococcus aureus* bacteremia confirmed with infective endocarditis and without infective endocarditis.**

| **Variables** | | **Endocarditis group**  **(*n* = 92)** | **Non endocarditis group**  **(*n* = 234)** | **P-value** |
| --- | --- | --- | --- | --- |
| **Methicillin-resistant *S*. *aureus*** | | 47 (51.1%) | 101 (43.2%) | 0.196 |
| **Male** | | 53 (57.6%) | 149 (63.7%) | 0.310 |
| **Community-onset infection** | | 64 (69.6%) | 172 (73.5%) | 0.474 |
| **Community-acquired infection** | | 22 (23.9%) | 52 (22.2%) | 0.743 |
| **Cardiac prosthesis** | | 12 (13.0%) | 4 (1.7%) | <0.001 |
| **Bone or joint infection** | | 11 (12.0%) | 72 (30.8%) | <0.001 |
| **Prolonged bacteremia** | | 26 (28.3%) | 71 (30.3%) | 0.711 |
| **SOFA score** ≥ **6** | | 43 (46.7%) | 60 (25.6%) | <0.001 |
| **Primary site of infection** | |  |  |  |
|  | Primary bacteremia | 10 (10.9%) | 34 (14.5%) |  |
|  | Central line-associated infection | 14 (15.2%) | 34 (14.5%) |  |
|  | Endovascular other than endocarditis | 11 (12.0%) | 23 (9.8%) |  |
|  | Infective endocarditis | 31 (33.7%) | 0 |  |
|  | Vertebral osteomyelitis | 5 (5.4%) | 36 (15.4%) |  |
|  | Bone or joint infection other than vertebral osteomyelitis | 6 (6.5%) | 36 (15.4%) |  |
|  | Skin and soft tissue infection | 6 (6.5%) | 40 (17.1%) |  |
|  | Lung | 1 (1.1%) | 11 (4.7%) |  |
|  | Others | 8 (8.7%) | 20 (8.5%) |  |

Note. SOFA, sequential organ failure assessment

| **S-Table 2. Physiological properties of *Staphylococcus aureus* isolated from patients with or without endocarditis, based on binding ratio *vs.* phosphate-buffered saline control.** | | | |
| --- | --- | --- | --- |
| **Physiological property** | **Endocarditis group**  **(*n* = 37)** | **Non endocarditis group**  **(*n* = 37)** | **P-value** |
| **Fibrinogen binding ratio** | 2.39 (± 0.80) | 2.25 (± 0.86) | 0.333 |
| **Fibronectin binding ratio** | 2.88 (± 0.93) | 2.34 (± 0.69) | 0.014 |
| **Internalization (× 10^6^ CFU/mL)** | 7.0 (± 9.4) | 6.8 (± 7.7) | 0.665 |

Note. CFU, colony forming unit

**Supplementary data**

**Clonality of isolates**

**Cluster analysis by clonal complex analysis and spa typing**

Clonal complex and spa type analyses were performed using multi-locus sequence type methods (Enright MC, Day NP, Davies CE, Peacock SJ, Spratt BG. Multilocus sequence typing for characterization of methicillin-resistant and methicillin-susceptible clones of *Staphylococcus aureus*. J Clin Microb **2000**; 38(3): 1008-15.)

Clonality of enrolled isolates was assessed by spa typing and multilocus sequence typing (MLST). In methicillin-susceptible *S*. *aureus* (MSSA), CC8 (*n* = 8, 20.0%), CC188 (*n* = 8, 20.0%), and CC1 (*n* = 5, 12.5%) were frequently observed. Among methicillin-resistant *S*. *aureus* (MRSA) isolates, CC5 (*n* = 18, 52.9%) and CC8 (*n* = 15, 44.1%) were the most commonly observed groups. The proportion of IE cases caused by each clonal complex did not differ between MSSA and MRSA isolates.

The strains used in this study were collected from 10 hospitals and are very unlikely to be related based on the genetic profiles of the isolates. Additional verification by clonal complex analysis and spa type analysis was used to screen for potential relationships between isolates, with none showing any genetic association or possibility of outbreak. However, the numbers of strains that were related to IE, such as CC5, CC30, and CC45, were similar in IE and non-IE isolates, making it difficult to confirm the frequencies of occurrence of IE due to clonal complexes.
